# Stomachs

**Published:** 1876-01

**Authors:** 


### STOMACHS.

We have seen folks, in fact we meet them daily, who seem to have no stomachs; while others have that viscus entering largely into their composition. We envy the chap that has no stomach; what a big, fat, good-natured looking fellow he is! It matters not to him, where or *what* he eats, much less the quantity that is taken into his capacious maw. The cyclopean faculty for using the organs of deglutition in many persons, is simply bewildering. Why, we have a friend,—a banker, who can sit at his desk all day, dispatch an incalculable amount of business, and then, without any more physical exertion than is necessary to carry him to his table, two squares away, eat two quail, a partridge, a quart of mushrooms, one dozen olives, and while he quaffs his mug of ale, or bottle of claret, disposes of a piece of pie and a quantity of brandy cheese; and, notwithstanding his pulse records one-hundred and ten beats to the minute, that friend is able to smoke two outrageously strong cigars, and in the innocence of his enjoyment, still lives. "But," observes the medical man, "That person cannot live long, with such habits and such a pulse." Well, perhaps not, but that does not lessen the wonderful capabilities of his stomach, and as that is the subject in hand, we pronounce it astounding, and would give a handsome sum for its possession, by any other means than a *post mortem.*

We have known individuals to eat, at one breakfast, a stack of pan-cakes twelve high, interlarded with melted butter and dripping in maple syrup, —a large dish of fried potatoes,—a quantity of fried ham and eggs, washing it all down with a cup of coffee; and, after smoking three or four cigars, imagine they had tape worms, because of an intermittent pulse, and a constricted feeling in the region of the stomach. We now have in mind a person who leads a life of unusual activity, exerting his nervous force to its utmost, who is slight and frail, with sufficient strength to do about half the mental and physical labor which he has assumed, and who withal is a confirmed dyspeptic, and will sit down to his meals, eating as much as would satisfy two ordinary mortals, wash it down with hard cider, and then be surprised that his food does not "lay well upon his stomach." Then, we've known a gentleman who came home late at night, ate two or three pickled pig's feet, half a mince pie, drank a bottle of scotch ale, and topped off by smoking a large pipe of tobacco before going to bed. It is needless to add that he is *all* stomach now, but cannot digest a huckleberry, and lives on oat meal porridge and other baby food.

In looking for a good-natured, jolly sort of a man, always select one with no stomach, or one who is not conscious of having any. Watch him for a few years, and when he becomes crabbed, morose, and commences swallowing gin and tansy, or brandy and water, to relieve his aches, be assured he has finally come to it—he's got a stomach, has just found it out, and from this time on, he becomes proportionately miserable to the amount of lager, ale, gin, or brandy he swallows in order to strengthen that confounded stomach.

In view of our experiences in this matter, we pronounce stomachs in general, and in particular, infernal nuisances, devised and arranged for man's torment and torture, for when a fellow has dyspepsia, he has *everything* in the calendar of woes, from cancer down, or up, to heart disease, liver complaint, and consumption! Should we be permitted to have a voice in the selection of our digestive organs, in the next world, we shall select a porcelain-lined copper retort, and have it occupy the usual position, and perform all the functions of the *stomach.*

---

☞Envelope and blank in this number. Send your 55 cents.

